# Tyrosine Kinase Self-Phosphorylation Controls Exopolysaccharide Biosynthesis in *Gluconacetobacter diazotrophicus* Strain Pal5

**DOI:** 10.3390/life11111231

**Published:** 2021-11-13

**Authors:** Katyanne Wanderley, Dayse Sousa, Gabriel Silva, Josemir Maia, Maria Silva, Marcia Vidal, José Baldani, Carlos Meneses

**Affiliations:** 1 Programa de Pós-Graduação em Ciências Agrárias, Campus I, Universidade Estadual da Paraíba, Campina Grande 58429-500, Brazil; kathy.maciel08@gmail.com (K.W.); dayse.freitas.sousa@aluno.uepb.edu.br (D.S.); jmouram@servidor.uepb.edu.br (J.M.); 2 Centro de Ciências Biológicas e da Saúde, Departamento de Biologia, Campus I, Universidade Estadual da Paraíba, Campina Grande 58429-500, Brazil; gabriel.sousa.silva@aluno.uepb.edu.br (G.S.); mazeuepb@servidor.uepb.edu.br (M.S.); 3 Embrapa Agrobiologia, Rodovia BR 465, km 07, Seropédica 23891-000, Brazil; marcia.vidal@embrapa.br (M.V.); ivo.baldani@embrapa.br (J.B.)

**Keywords:** plant growth-promoting bacteria, exopolymer production, bacterial protein phosphorylation, autophosphorylation

## Abstract

The biosynthesis of exopolysaccharides (EPSs) is essential for endophytic bacterial colonisation in plants bacause this exopolymer both protects bacterial cells against the defence and oxidative systems of plants and acts on the plant colonisation mechanism in *Gluconacetobacter diazotrophicus*. The pathway involved in the biosynthesis of bacterial EPS has not been fully elucidated, and several areas related to its molecular regulation mechanisms are still lacking. *G. diazotrophicus* relies heavily on EPS for survival indirectly by protecting plants from pathogen attack as well as for endophytic maintenance and adhesion in plant tissues. Here, we report that EPS from *G. diazotrophicus* strain Pal5 is a signal polymer that controls its own biosynthesis. EPS production depends on a bacterial tyrosine (BY) kinase (Wzc) that consists of a component that is able to phosphorylate a glycosyltranferase or to self-phosphorylate. EPS interacts with the extracellular domain of Wzc, which regulates kinase activity. In *G. diazotrophicus* strains that are deficient in EPS production, the Wzc is rendered inoperative by self-phosphorylation. The presence of EPS promotes the phosphorylation of a glycosyltransferase in the pathway, thus producing EPS. Wzc-mediated self-regulation is an attribute for the control of exopolysaccharide biosynthesis in *G. diazotrophicus*.

## 1. Introduction

The use of microbial inoculants with biofertiliser and biostimulant properties has emerged as an alternative to replace or complement the use of synthetic fertilisers to promote plant growth and development. As a result, the environmental liabilities of agricultural practices are reduced. Bio-based agricultural inputs and technologies also represent tools for increasing national sovereignty in the face of the challenges of producing food, fibres, and energy in the world.

*Gluconacetobacter diazotrophicus* is a plant growth-promoting bacteria (PGPB) that can be found colonising the internal tissues of many plants of agricultural importance, such as sugarcane (*Saccharum officinarum*) [1], rice (*Oryza sativa*) [2] and the C_4_- energy crop, and elephant grass (*Pennisetum purpureum*) [3], in high numbers. In addition to fixing nitrogen in association with sugarcane plants, it has been demonstrated that this bacterium has other interesting physiological abilities, such as the production of indole acetic acid [4] and siderophores [5] and the solubilisation of inorganic phosphate and zinc oxide [6]. Its ability to control sugarcane phytopathogens, such as *Colletotrichum falcatum* and *Xanthomonas albilineans* [7], has also been demonstrated. *G. diazotrophicus* strains tolerate high concentrations of sucrose (10%) and low pH, suggesting the existence of highly efficient mechanisms for protection against acidity and osmotolerance [8]. Nitrogenase activity is inhibited by high concentrations (20 mM) of ammonium sulphate, ammonium chloride, and glutamate [9], whereas amino acids (asparagine, aspartic acid and glutamic acid) partially inhibit this activity [10].

It has been reported that some Gram-negative species can synthesise different exopolysaccharides (EPSs) and that Wzc/Wzb-dependent regulatory proteins are also involved in the synthesis of different cell-surface polysaccharides [11,12]. In general, Wzc contains amino acid sequences that resemble Walker A (GXGK[T/S]) and Walker B (hhhD), where h represents a hydrophobic amino acid motif that is normally found in nucleotide-binding proteins but not in eukaryotic tyrosine kinases [12]. A tyrosine cluster (YC) containing multiple tyrosine residues is also located at the C-terminal end of Wzc; this cluster varies in length (10 to 25 amino acids) and contains several tyrosine residues (five to ten) that constitute the self-phosphorylation sites of Wzc. In addition to these two motifs, there is also the Walker A lysine, which, according to some studies, induces a strong decrease in the autokinase activity of Wzc [12]. Finally, a tyrosine at position 569 stimulates the self-phosphorylation of the tyrosine [13].

Bacteria of the genus Gluconacetobacter have been shown to produce large amounts of EPS. In *Gluconacetobacter xylinus*, bacterial cellulose is biosynthesised from sugars and organic acids. In addition to insoluble cellulose, *G. xylinus* can also produce a water-soluble EPS, named acetan, which has a backbone of [→4)-β-d-Glcp-(1→]_n_ branched with a penta- or tetrasaccharide containing Man, Glc, Rha, and GlcA [14]. Within the species *G. diazotrophicus*, strain Pal5 has been reported to produce a single EPS type, and it has been demonstrated that this EPS structure, which lacks deoxy-hexoses and acidic units, shows 4-O-substituted glucopyranosyl residues in appreciable amounts. It has also been demonstrated to have a 2-O-linked-α-mannose unit, and those C6-linked units were found to be more labile and were, therefore, the cleaving point in the structure, which yielded a pentasaccharide [15].

A series of experiments involving the MGD mutant (Table 1) in *G. diazotrophicus* Pal5 indicated that the functional gumD gene (glycosyltransferase gene) is involved in EPS biosynthesis, biofilm formation on glass wool, rice root surface colonisation, and the endophytic colonisation of rice seedlings [16]. Using a mutagenesis approach, we demonstrated that the gumD gene of *G. diazotrophicus* Pal5 is involved in EPS production. It has been shown that a successful endophytic colonisation of rice plants by the *G. diazotrophicus* strain Pal5 involves two phases: first, there is an adsorption phase that is mediated by flagella and other proteins followed by an anchoring phase, which requires the presence of EPS [16]. Similar EPS activity has been found in several bacterial genera that interact with plants, such as *Azoarcus, Rhizobium, Azospirillum, Sinorhizobium, Paraburkholderia,* and *Bradyrhizobium* [17,18,19].

The complete sequencing of the genome of *G. diazotrophicus* strain Pal5 [20] has opened new perspectives for understanding the function of many unknown genes. Transposon mutagenesis has already been applied to *G. diazotrophicus* strain Pal5 and has allowed the selection of several mutants with different impaired genes and diverse phenotypes [20,21].

The production of EPS by the *G. diazotrophicus* strain Pal5 requires 15 genes, which are clustered in one region on the chromosome [22]. However, the function of each gene in this cluster still requires more detailed studies. Research involving *Escherichia coli* (colanic acid), *Xanthomonas campestris* (xanthan), several *Sphingomonas* and *Pseudomonas* strains, several *Rhizobium*, *Agrobacterium*, *Alcaligenes*, and *Pseudomonas* strains (succinoglycan), *Enterobacteria* strains (sphigan), *Sphingomonas* sp. ATCC 53159 (diutan), *Sphingomonas elodea* ATCC 31461 (gelan), and *Alcaligenes* sp. CGMCC2428 (welan) have shown that EPS biosynthesis takes place in a pathway that is dependent on two regulatory proteins: Wzc-tyrosine kinase and Wzb-phosphatase [23]. In the Wzc/Wzb-dependent regulatory pathway in *Escherichia coli*, the assembled O-antigen repeat units are translocated from the cytosolic to the periplasmic face of the inner membrane by a Wzc translocase and are then polymerised by the integral membrane protein Wzc to form a celullose chain [24]. In the *G. diazotrophicus* strain Pal5, analyses of the ORFs GDI2535 and GDI2549 have shown sequence homology with the low molecular weight protein tyrosine phosphatase and the bacterial tyrosine (BY) kinase [23], where polysaccharides synthesised via Wzc/Wzb have high sugar pattern diversity. The bacteria that use this system are known to contain genes for phosphatase (*wzb*) and the BY kinase (*wzc*) within extracellular polysaccharide operons [23].

Here, we report that the extracellular domain of the membrane component Wzc (GDI2549), a BY kinase, is a receptor that directly recognises EPS. We propose that the BY kinase activity in *G. diazotrophicus* strain Pal5 mediates a previously unrecognised and perhaps widespread system of intercellular communication that controls exopolysaccharide production.

## 2. Materials and Methods

### 2.1. Annotation of the Putative Wzc Protein

The sequence of the *G. diazotrophicus* strain Pal5 *wzc* gene (locus GDI2549) is available in GenBank under accession number AM889285.1. Comparative (orthologue detection) and functional analyses of the Wzc of this bacterial genome were conducted using BLAST [25]. Pfam [26] and the enzyme nomenclature bank (Expasy Enzyme) [27] were used to confirm the results.

### 2.2. Bacterial Strains and Growth Conditions

*G. diazotrophicus* strain Pal5 (BR 11281, ATCC49037) was obtained from the Diazotrophic Bacteria Culture Collection of Embrapa Agrobiologia, Brazil. *G. diazotrophicus* wild type and mutant strains were grown in LGIP [1] liquid media at 30 °C. The C2 liquid medium [28] was used to prepare electrocompetent Pal5 cells. The antibiotics kanamycin (200 μg/mL) and/or ampicillin (500 μg/mL) were added as required. For cloning purposes, *E. coli* DH10B cells were cultivated in Luria–Bertani (LB) medium [29] that had been amended with the selective antibiotics kanamycin (50 µg/mL) or ampicillin (100 µg/mL). For EPS determination in the culture supernatants, bacterial cells were grown in LGI medium [1] modified by Meneses [16]. Where indicated, purified exogenous EPS from wild-type Pal5 strain (100 µg/mL) was also added. The strains and primers used in this study are listed in Table 1.

In this work, the following strains of *G. diazotrophicus* were used: Pa5, wild-type; MGD, defective in EPS production and with the gumD gene knocked out; MWzc, defective in EPS production and with the *wzc* gene (GDI2549) knocked out; ΔW_Pal5, *E. coli* DH10B heterologously expressing the *G. diazotrophicus* Pal5 *wzc* gene; ΔW_MGD, *E. coli* DH10B heterologously expressing the *G. diazotrophicus* MGD *wzc* gene; and ΔW_Wzc, *E. coli* DH10B heterologously expressing the *G. diazotrophicus* MWzc *wzc* gene.

### 2.3. Construction of Wzc Mutant of G. Diazotrophicus

The complete coding sequence of the Pal5 *wzc* (GDI2549) orthologue was amplified by PCR with the primers *wzc*-sense and *wzc*-antisense (Table 1), using Taq polymerase (Invitrogen, Paisley, UK). The 730-bp PCR product was cloned into the pGEM-T easy vector (Promega Corp., Madison, WI, USA). One recombinant plasmid containing the desired fragment (pWzc) was selected and mutagenised in vitro by the insertion of the commercial *EZ::Tn5 < KAN-2 >* Transposon (Tn5) (EpicentreTechnologies Corp., Madison, WI, USA) following the instructions of the manufacturer. Putative transformants were selected on Dygs plate medium containing the selective antibiotics kanamycin (200 μg/mL) and/or ampicillin (500 μg/mL). The Wzc defective strain was named MWzc.

### 2.4. EPS Purification and Quantification

The production of EPS by the *G. diazotrophicus* wild-type and mutant strains was evaluated after bacterial growth in 50 mL of modified LGI medium with mannitol as a carbon source (20 g/L) in an orbital shaker (200 rpm, 30 °C) for 72 h. The EPS present in the culture supernatants was precipitated with ethanol and was then dissolved in MilliQ H_2_O [30]. The EPS production was determined by quantifying the total carbohydrates using the colorimetric phenol sulphuric method as described [31].

### 2.5. Tyrosine Kinase Assay with Radiolabeled ATP

Wild-type Pal5 and MGD and MWzc mutants cells were treated with hydralazine for 2 h. Cells were lysed in lysis buffer (50 mM HEPES, pH 7.7, 1.5 mM MgCl_2_, 0.2 mM Na_3_VO_4_, 1 mM EGTA, 150 mM NaCl, 10% Glycerol, 100 mM NaF, 50 mM beta Glycerophosphate, 0.1% NP40) containing a protease inhibitor cocktail via sonication 2x for 10 s. Cell debris was removed by centrifugation (15,000 × g, 10 min) at 4 °C, and supernatants were used for tyrosine kinase assays after protein measurement. Reactions were initiated by adding 20 μg histone 2B (protein substrate), 50 μM ATP ([γ−32P] ATP 10,000 cpm/pmol) (MP Biomedicals, Valiant Co., Yantai, Shandong, China), 10 mM MgCl_2_, 10 mM HEPES pH 8.0, 1 mM DTT, and 1 mM benzamidine for 1 h at room temperature [26]. The reaction was stopped by adding 5x Laemmli sample buffer followed by heating at 100 °C. Samples were loaded on 4–12% Crit XT Bis-Tris gels (Bio-Rad, Hercules, CA, USA). Gel staining, drying, and scintillation counting were performed [32]. The histone 2B band from the lane that lacked cell extract was subtracted to correct for background. In cMWzc and cMGD, purified exogenous EPS (100 μg/mL) was also added.

### 2.6. In Vitro IP Kinase Assay

The molecular dynamics of the tyrosine kinase activity (Wzc) were evaluated after the strains had grown. To do that, 500 μL of cultured cells of each strain were washed three times in sterile saline solution. Soon after, the cells were resuspended in modified LGI medium, and, in this case, there was an exchange of K_2_HPO_4_ and KH_2_PO_4_ by radioactively labelled γ−32P orthophosphate. Strains were grown in the presence of γ−32P orthophosphate for 12 h, and the results were detected by immunoprecipitation. To prepare the total lysates, the cells were lysed in PBS buffer pH 7.4 containing 1% Triton X-100 protease and phosphatase inhibitor cocktail ((pH 7.4 and containing 1% Triton X-100 and 200 μg/mL Lysozyme (Sigma-Aldrich^®^, St. Louis, MO, USA), a cocktail containing protease and phosphatase inhibitors (1:10, Sigma-Aldrich^®^, individual components and concentration: aprotinin (bovine lung): serine protease inhibitor (0.08 mM); bestatin: aminopeptidase B and leucine aminopeptidase inhibitor (5 mM); E-64: cysteine protease inhibitor (1.5 mM); leupeptin hemisulfate: serine/cysteine protease inhibitor (2 mM); β-Glycerophosphate: serine/threonine phosphatase inhibitor (10 mM); sodium fluoride: acid phosphatase and serine/threonine phosphatase inhibitor (50 mM); sodium orthovanadate: protein tyrosine phosphatase and alkaline phosphatase inhibitor (1 mM); sodium pyrophosphate decahydrate: serine/threonine phosphatase inhibitor (10 mM); and EDTA disodium salt: metalloprotease inhibitor (0.5 mM)). Lysates were passed through a 1 mL 26G ½ syringe three times and were then frozen (−80 °C) and thawed three times. The lysates were further sonicated for 30 min at an amplitude of 60 Hz (Branson Sonifier 250, Hielscher, Teltow, Germany) with the probe in an ice bath.

The pre-clearing of the lysates was conducted by incubating the samples with 30 µL of protein G-agarose beads (Life Technologies, Carlsbad, CA, USA) that had been previously washed with PBS buffer pH 7.4 for two hours at 4 °C. After centrifugation at 100× *g* for two minutes at 4 °C, the supernatant containing the soluble protein lysate was carefully removed to be used in the immunoprecipitations. Then, 50 μL of protein G-agarose beads were washed and incubated with antibodies (5 μL of anti-Wzc antibody). Due to the use of monoclonal antibodies, 5 μg of rabbit anti-IgG antibody was added and shaken, and the reaction was incubated for a further two hours at 4 °C under constant agitation. After that, the beads were washed three times with PBS pH 7.4 to remove any unbound antibodies. Then, the beads were blocked with PBS pH 7.4 containing 1 mg/mL BSA for 30 min at 4 °C. For the next step, the protein G-agarose beads containing bound anti-GDIWzc antibodies were incubated with the cell lysates for 16 h at 4 °C under constant agitation. After the incubation of the samples, the beads were washed by centrifugation three times with PBS pH 7.4 containing the protease and phosphatase inhibitor cocktails. To elute the proteins that had bound to the antibodies, the beads were resuspended in Laemmli buffer [33], heated at 100 °C for ten minutes, and analysed as per the previous item.

### 2.7. Cloning and Expression of the Wzc Gene in E. coli Strain BL21-AI

Specific PCR primers were designed for the *wzc* gene (GDI2549) from *G. diazotrophicus* strain Pal5 and the MGD and MWzc mutants (Table 1). The *E. coli* expression system with Gateway^®^ technology (Thermo Fisher Scientific, Waltham, MA, USA) was applied to express the Wzc heterologous protein in *E. coli* strain BL21-AI™ using the suitable plasmids [34,35] and quantified [36]. The functionality of Wzc in different strains from *G. diazotrophicus* was tested by using heterologous expression in *E. coli* strain BL21-AI™. The *E. coli* strain BL21-AI™ was transformed, and the recombinant strains ΔW_Pal5, ΔW_MGD, and ΔW_MWzc were obtained and heterologously expressed His6-tagged Wzc.

### 2.8. RT-qPCR Analysis

*G. diazotrophicus* Pal5, MGD, and MWzc strains were grown in LGI medium supplemented with different individual carbon sources (glucose, sucrose, fructose, mannitol; 20 g/L, and exogenous EPS; 100 μg/mL). Cultures were maintained at 30 °C and at 200 rpm for 72 h. When the cultures reached a growth stage of approximately 6.0 × 10^7^ cells/mL, the cells were centrifuged at 4000× *g* for 5 min, and the bacterial precipitate was used for total RNA extraction.

Total RNA was isolated with Trizol, according to the manufacturer’s protocols. RNA purity and integrity that were appropriate for downstream RT-qPCR applications were confirmed through measurement with a Qubit 4 Fluorometer (Thermo Fisher Scientific, Waltham, MA, USA). After DNase I treatment, first-strand cDNA synthesis was conducted using the Superscript^®^ IV Master Mix (Invitrogen, Thermo Fisher Scientific, Waltham, MA, USA), which was performed according to the manufacturer’s manual. The RT-qPCR reactions were conducted in a StepOnePlus Real-Time PCR thermocycler (Life Technologies, New York, NY, USA). Each reaction contained 12.5 μL Power SYBR Green PCR Master Mix (Applied Biosystems, Waltham, MA, USA); 0.4 μL (1 mM) of each specific oligonucleotide (RTwzc, Table 1), as was also the case with the endogenous control gene (23S, Table 1); appropriate amounts of cDNA (1 μL, 1:4 dilution); and 10.7 μL PCR water to yield a final volume of 25 μL. The qPCR reactions were performed under the following conditions: initial denaturation at 95 °C for three min followed by 40 cycles of 95 °C for 30 s, 60 °C for 30 s, and a final extension at 60 °C for 5 min. Bacterial cells grown in LGI medium were used to normalise the data. The 2^−ΔΔCT^ method was applied to quantify the relative cDNA gene expression, and the *23S rRNA* gene was used as an internal reference control [37]. The results are the average of six levels of replication (three biological replications and three technical replications for each biological replication).

### 2.9. Statistical Analysis

The results are presented as means with standard deviation and were evaluated by one-way statistical analysis of variance followed by the Tukey test. All experiments were performed in biological and technical triplicates and were analysed by SigmaPlot 11.0 software (Systat Software, Chicago, IL, USA). In all cases, the differences were considered significant at *p* < 0.05.

## 3. Results

### 3.1. Homology of the Strain Pal5 Wzc Protein to Other Bacterial Proteins

**Figure 1:**
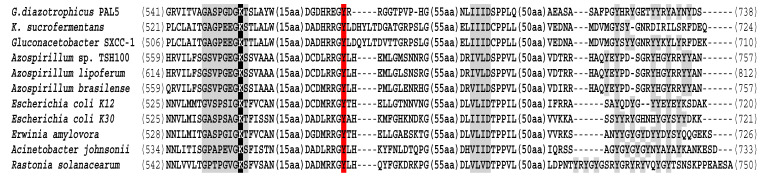
Partial multiple alignment of the conserved C-terminal region of predicted *G. diazotrophicus* Wzc protein sequence with those assigned from proteins such as BY kinases. Walker A, B, and Tyrosine cluster (grey), catalytic lysine of Walker A motif (black), and Tyrosine_585_ residue (red) are noted.

An analysis of the Wzc protein from strain Pal5 compared to other bacterial species with a protein found in Genbank showed that it had 92% similarity (individual gene products) to the cluster from *Komagataeibacter sucrofermentans*. Furthermore, high (90%) similarity was also found to a protein from the phylogenetically closely related endophyte *Gluconacetobacter* SXCC-1 and to the diazotrophic *Azospirillum* species *A. lipoferum* (87% similarity) and *A. brasilense* (84% similarity). Therefore, based on the presence of this protein homology and organisation, the EPS produced by strain Pal5 is most probably regulated by the Wzc/Wzb system (Figure 1).

Gram-negative bacterial Wzc proteins are generally large (~80 kDa) and composed of an N-terminal transmembrane domain and a C-terminal Wzc domain [38] containing the Walker A and B active sites (Figure 1). In *G.diazotrophicus* Pal5, we identified a protein with high similarity to Wzc from the TY Kinase family that has a molecular mass of 78.1 kDa and possessing well-characterised functional domains for this family of proteins. As has been described for *E. coli* K12 [38], intermolecular self-phosphorylation in the C-terminal tyrosine cluster (five tyrosine’s from position 708 to position 715) should be stimulated by self-phosphorylation at Tyr_569_, where it can be deduced that in *G. diazotrophicus* happens in Tyr_585_.

### 3.2. Tn5 Insertional Inactivation of the PAL5 Wzc Gene

To investigate the role of Wzc in EPS biosynthesis by Pal5, the *wzc* gene was interrupted by the insertion of a commercial Tn5 transposon, generating the mutant MWzc. This mutant was molecularly validated by Southern blot analysis and reverse polymerase chain reaction (IPCR) followed by sequencing, which confirmed that the insertion of Tn5 occurred after position 1755 of the base pair of the *wzc* gene codon encoding uma tyrosine at position 585, which stimulates the self-phosphorylation of the tyrosine in Wzc from other bacteria [13].

### 3.3. EPS Production Is Impaired in a Wzc Mutant

**Figure 2:**
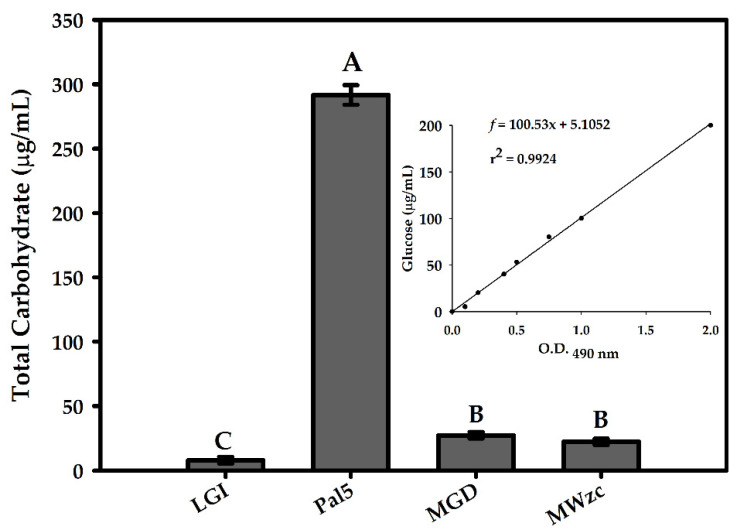
Quantification of exopolysaccharide (EPS) production by the wild-type strain Pal5 and mutants MGD and MWzc grown in LGI medium. LGI medium; wild-type Pa5; MGD, defective in EPS production and with the *gumD* gene knocked out; and MWzc, defective in EPS production and with the *wzc* gene (GDI2549) knocked out. Bars represent the standard deviation (*n* = 3). Different letters indicate statistical difference (Tukey’s test, *p* < 0.05). Inset: EPS from culture supernatants was precipitated with ethanol and dissolved in water; then, the total carbohydrates were quantified by the phenol sulphuric method (calibration curve).

The in vitro production of EPS by *G. diazotrophicus* wild-type and mutant strains was investigated. To do this, the total carbohydrates precipitated from the culture supernatants were quantified and assumed to represent the total EPS produced under the growth conditions. The results showed that the highest quantities of EPS were produced by the wild-type strain Pal5 when compared to the control with the LGI medium (Figure 2).

In contrast, the production of EPS by the MGD and MWzc mutants was almost eliminated, considering that the EPS level was reduced by 90% in the MGD and 94% in the MWzc mutants. These results indicate that, as expected, Pal5 (wild type) naturally produces EPS; MGD [16] was defective in EPS production and had the gumD gene knocked out; MWzc was also defective in EPS production and had the *wzc* gene (GDI2549) knocked out.

### 3.4. Glycosyltransferase Phosphorylation in G. diazotrophicus

**Figure 3:**
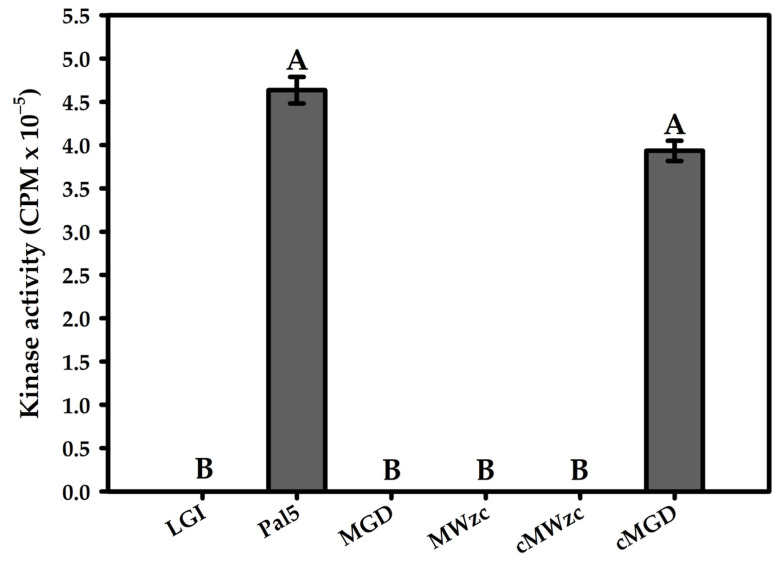
Detection of the glycosyltransferase phosphorylation. LGI medium; wild-type Pa5; MGD, defective in EPS production and with the *gumD* gene knocked out; MWzc, defective in EPS production and with the *wzc* gene (GDI2549) knocked out; cMWzc was also defective in EPS production and with the *wzc* gene (GDI2549) knocked out and complemented with exogenous EPS from *G. diazotrophicus* Pal5; and cMGD, defective in EPS production and with the *gumD* gene knocked out and complemented with exogenous EPS from *G. diazotrophicus* Pal5. Absolute quantification of the scintillation emitted by different strains. Bars represent the standard deviation (*n* = 3). Different letters indicate statistical difference (Tukey’s test, *p* < 0.05).

After the in silico characterisation of the protein encoded by the *wzc* (GDI2549), an assay was performed to detect its kinase activity in vivo (Figure 3). To test whether Wzc can phosphorylate glycosyltransferases, we used defective mutant strains in the production of EPS. The cells grown under EPS-inducing conditions (as described in item Section 2.4) were collected to determine the phosphorylation state of the glycosyltransferases, as shown in the scintillation analysis (Figure 3). The results demonstrated that the protein was able to phosphorylate the substrate (γ−32P orthophosphate) used in the assay in the presence of EPS, thus indicating its BY kinase function as predicted by the in silico analysis. It is also worth noting that in addition to the kinase activity of MGD, which is conditioned by the presence of exogenous EPS, the results showed that the presence of exogenous EPS was able to promote the BY kinase activity.

The MWzc mutant strain supplemented with exogenous Pal5 EPS (cMWzc) cells confirmed that the glycosyltransferases were phosphorylated (Figure 3), thus indicating that the glycosyltransferases had undergone phosphorylation per the tyrosine residues (in cMWzc, purified exogenous EPS (100 μg/mL) was also added). Decreased counts per minute (CPM) were observed in the LGI pure medium, MGD, MWzc mutants, and cMWzc exposed to scintillation (100%) due to the total absence of EPS. However, the application of exogenous EPS promoted a decrease of only 11.1% (not a statistically significant difference).

### 3.5. Immunoprecipitation Assay for Detection of Protein Tyrosine Kinase Self-Phosphorylation Dynamics with Radiolabeled ATP

**Figure 4:**
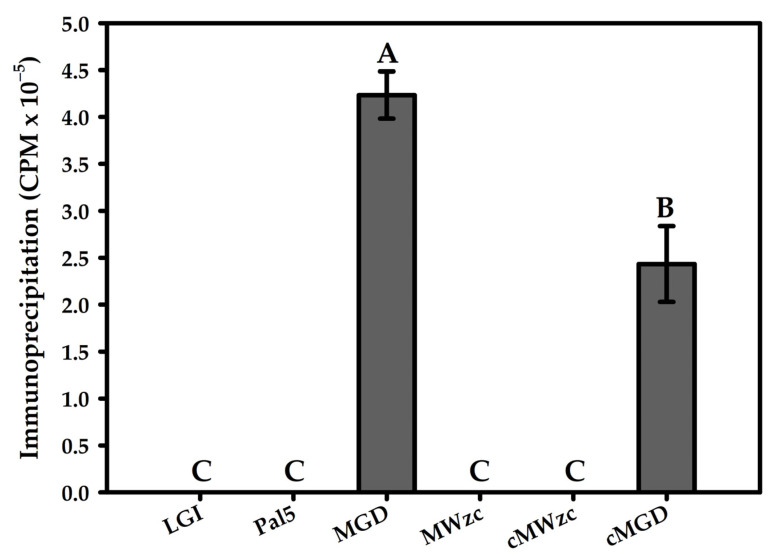
Immunoprecipitation indicating Wzc self-phosphorylation in MGD and MWzc mutants. LGI medium; wild-type Pal5; MGD, mutant defective in EPS production with the *gumD* gene knocked out; MWzc, mutant defective in EPS production with the *wzc* gene (GDI2549) knocked out; cMWzc was also defective in EPS production and with the *wzc* gene (GDI2549) knocked out and complemented with exogenous EPS from *G. diazotrophicus* Pal5; and cMGD, defective in EPS production and with the *gumD* gene knocked out, supplemented with exogenous EPS from *G. diazotrophicus* Pal5. Absolute quantification of the scintillation emitted by wild-type strain Pal5 and by mutants deficient in exopolysaccharide production and by the cMWzc and cMGD complemented with exogenous EPS. Bars represent the standard deviation (*n* = 3). Different letters indicate statistical difference (Tukey’s test, *p* < 0.05).

To determine whether this phosphorylation was indeed dependent on the kinase activity of Wzc, we analysed the defective mutant strain (MWzc). Increased CPM (self-regulation) were observed in the MGD mutant and cMGD treatments exposed to scintillation (100%). There was a statistically significant difference with a decrease of 43.7% when the exogenous EPS was applied (Figure 4). The results obtained with the MGD mutant supplemented with 100 μg/mL of EPS purified from Pal5 suggests that the EPS interferes Wzc autophosphorylation activity in a dose-dependent manner.

Wzc self-phosphorylation was inactivated in Pal5 and in the MWzc mutant and cMWzc. Therefore, the presence of EPS seems to limit self-phosphorylation and, instead, promotes the phosphorylation of a glycosyltransferase, thus stimulating the production of exopolymers. Hence, these data strongly suggest that the *wzc* gene is involved in EPS production by strain Pal5 grown in vitro. Autoradiography analyses provided evidence that the absence of EPS promotes the self-phosphorylation of the Wzc protein both when it is and is not in the MGD mutant EPS supplement. Because the conditions between the treatments remained uniform for all of the samples, absolute measurements of the specific activity were sufficient to compare treatments. Stimulation by EPS results in the phosphorylation and activation of the glycosyltransferases, promoting more EPS synthesis and establishing a positive feedback loop.

### 3.6. Wzc Expression in E. coli BL21-AI

**Figure 5:**
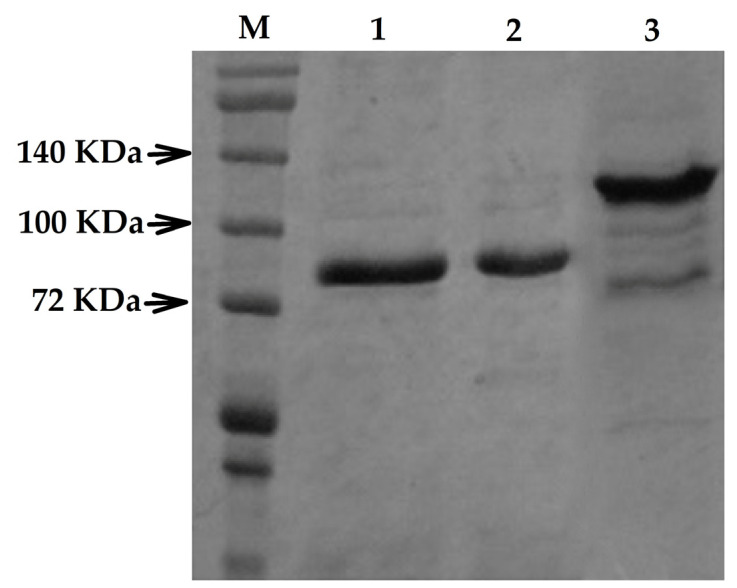
SDS-PAGE of His6-Wzc recombinants proteins. M: Molecular weight marker protein ladder (Cat No. 10747-012-Invitrogen); lane 1: ΔW_Pal5, *E. coli* DH10B heterologously expressing *G. diazotrophicus* Pal5 *wzc* gene; lane 2: ΔW_MGD, *E. coli* DH10B heterologously expressing *G. diazotrophicus* MGD *wzc* gene; and lane 3: ΔW_Wzc, *E. coli* DH10B heterologously expressing *G. diazotrophicus* MWzc *wzc* gene.

The heterologous expression of Wzc occurred at an expected band size of ~78.1 kDa for the ΔW_Pal5 and ΔW_MGD strains. However, the target protein was expressed at different levels in molecular mass for ΔW_Wzc, and this difference was due to the insertion of the Tn5 transposon that increased the mass of the knockout protein to ~130 kDa (Figure 5).

### 3.7. Wzc Gene Expression in Bacterial Strains Grown in Different Carbon Sources

**Figure 6:**
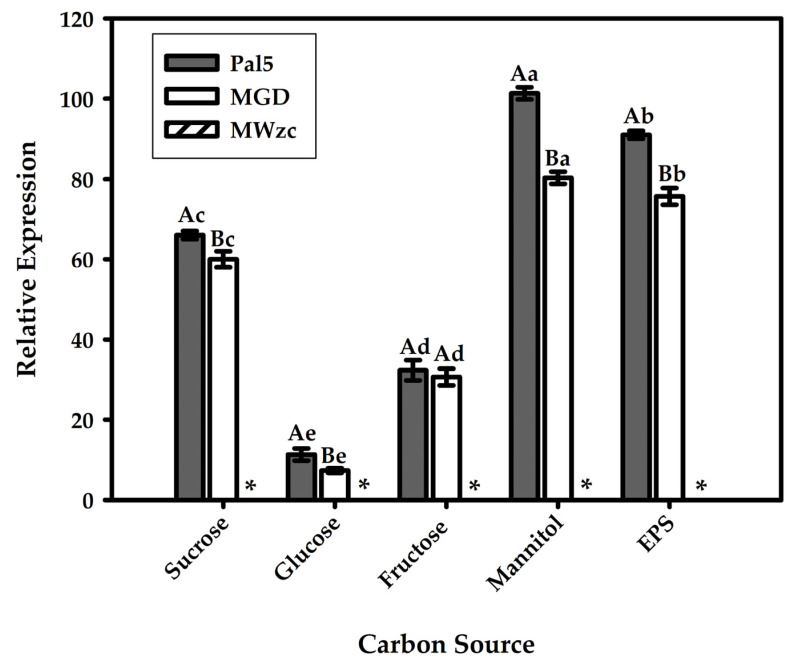
Relative expression of the *wzc* gene from *G. diazotrophicus* strain Pal5 involved in the regulation of exopolysaccharide biosynthesis. Wild-type Pal5; MGD, defective in EPS production and with the *gumD* gene knocked out; and MWzc, defective in EPS production and with the *wzc* gene (GDI2549) knocked out. The transcript level is represented as the absolute value of the studied gene by the absolute value of the gene in the LGI medium growth condition (relative expression). Values were normalised in relation to the expression of the constitutive *23S* gene. Averages followed by identical capital letters for the same carbon source do not differ among the *G. diazotrophicus* strains (Pal5, MGD and MWzc), and averages followed by identical lowercase letters for the same strain do not differ among the carbon sources. Bars represent the standard deviation (*n* = 3). * no relative expression detected.

To determine the individual contribution of the Wzc protein to exopolysaccharide production during the growth process, the relative expression of the *wzc* gene was monitored under different growth conditions: in LGI medium and in LGI medium supplemented with independent carbon sources: sucrose, glucose, fructose, mannitol (20 g/L), or exogenous EPS (100 μg/mL). The carbon sources were selected due to their good performance in the production of EPS of *G. diazotriphicus* Pal5, and sucrose was chosen in particular because it is the most abundant carbohydrate in plants [16] and in EPS purified from the supernatant of the Pal5 growth cultures (Figure 6).

The results showed an increase in the number of *wzc* transcripts in Pal5 and MGD for all of the treatments when compared to when they were grown in the LGI medium for 72 h, with the exception of the mutant strain (MWzc) grown in sucrose, glucose, fructose, mannitol, and EPS. It is noteworthy that the lowest relative expression value was observed for the treatment supplemented with glucose (11.3-fold and 10.4-fold; Pal5 and MGD, respectively). A generalized increase in the bacterial response with a 101.4-fold and 82.6-fold for Pal5 and MGD, respectively, in the expression of the *wzc* gene was observed when grown in the presence of mannitol and EPS. Interestingly, the relative expression of wzc in MGD was lower when compared to Pal5, where we suggest that this difference is caused by the amount of EPS that is produced, as shown in Meneses et al. [16].

## 4. Discussion

The attachment of plant growth-promoting bacteria (PGPB) to the plant root surface is the first step in enabling endophytic establishment. In these bacteria, EPS, along with flagella, pili, bacterial exudates, and signalling molecules, are important for the formation of biofilms and the first steps of the colonisation process. In addition, EPS contributes to the growth and survival of bacteria within the plant, helping the colonisation process and creating a favourable microenvironment where EPS may act as a barrier against plant defence mechanisms [16,39].

However, attachment to the root surface may not be sufficient for successful endophytic establishment, as demonstrated by Liu and associates [40], who discovered similar root-attachment capabilities for both exclusively rhizosphere-inhabiting and endophytic bacterial strains. Furthermore, Meneses et al. [39] proposed a mechanism for the antioxidant ability of EPS, wherein the antioxidant effect of polymers, which are significantly better free radical scavengers than monosaccharides, is independent of intrachain linkage, molecular weight, or degree of polymer branching. Based on the results of Sabra and associates [41], it is suggested that the production of EPS, particularly the formation of a biofilm on the cell surface, forms an effective barrier for O_2_ transfer into the cell, thus avoiding nitrogenase inactivation by O_2_. One of the key elements in the establishment and maintenance of the biofilm structure and properties is the extracellular matrix. This matrix is composed of water and EPS—primarily polysaccharides, proteins, and DNA. Characterisation of the matrix requires component identification as well as determination of the relative concentration of EPS constituents, including their physicochemical properties and descriptions of their interactions, in order to hypothesized the precise function of this extracellular matrix [16].

In the present study, the *wzc* gene from strain Pal5, hypothesised as being responsible for EPS production, was investigated using bioinformatic tools and laboratory assays. In general, the *wzc* gene from strain Pal5 was orthologous to the *wzc* gene from the phylogenetically closely related *K. sucrofermentans*. In addition, this gene was also similar to genes from phylogenetically distant plant-associated bacteria such as *Gluconacetobacter* SXCC-1, *A. lipoferum*, *A. brasilense* (diazotrophs), and *Rastonia solanacearum* (phytopathogenic species), suggesting a horizontal gene transfer mechanism. This speculation also corroborates the earlier studies on *S. pneumoniae*, *L. delbreuckii,* and *S. thermophilus* as well as those conducted in Gram-negative bacteria [42,43].

In *G. diazotrophicus* Pal5, Wzc consists of 738 amino acid residues with a calculated molecular mass of 78.1 kDa. Wzc contains amino acid sequences that resemble Walker A (GXGK[T/S]) and Walker B (hhhD). In addition, the *G. diazotrophicus* Pal5 Walker A (PXXGK) motif, which is important for Mg^2+^ binding, was located within the Wzc protein. A tyrosine cluster (YC) containing multiple tyrosine residues was located at the C-terminal end of Wzc; this tyrosine cluster is a long one (22 amino acids) and contains several tyrosine residues (seven) that constitute the self-phosphorylation sites of Wzc. In addition to these two motifs, we identified the presence of the Walker A lysine, which according to some studies, induces a strong decrease in the autokinase activity of Wzc. Finally, we identified a tyrosine *G. diazotrophicus* at position 585, which was able to stimulate the self-phosphorylation of the tyrosine.

Using a Tn5-mutagenesis approach, we demonstrated that the *wzc* gene of G. *diazotrophicus* strain Pal5 is involved in EPS production. Therefore, unmetabolised sugar is probably responsible for a large part of this precipitate. However, it is known that mannitol (carbon source) is soluble in water–ethanol mixtures to a limited extent [44]. Accordingly, the precipitate observed for the LGI pure medium and in the medium using mannitol as the carbon source for the MGD and MWzc mutants consisted of typical mannitol-like crystals, as previously described in similar experiments conducted by Meneses and associates [16] and Bouchard and associates [44].

**Figure 7:**
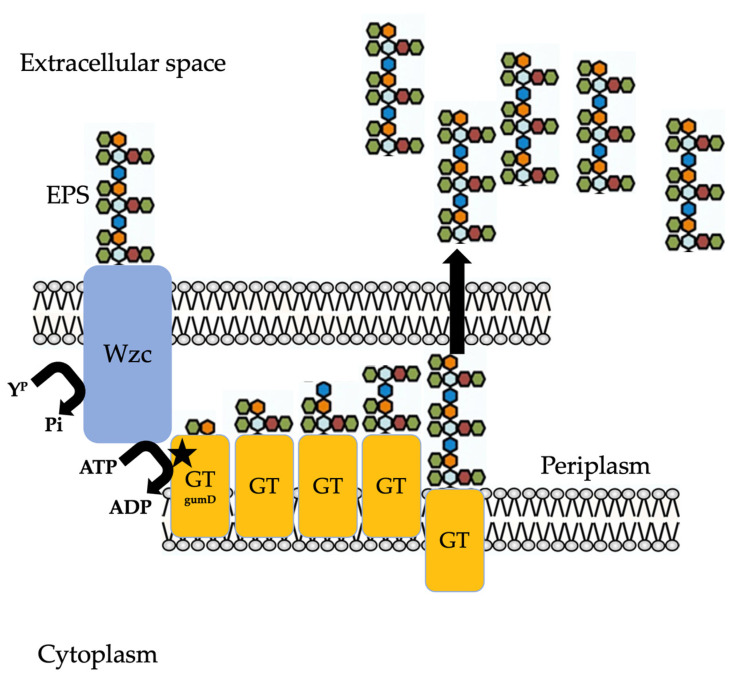
Models of EPS biosynthesis routes in *G diazotrophicus*, the Wzc-dependent. Glycosyltransferases (GTs) are periplasmatic, whereas Wzc is an outer membrane protein. Polymerization requires a Wzc. EPS, exopolysaccharides; ATP, adenosine-5′-triphosphate; ADP, adenosine-5′-diphosphate; YP, phosphorylated tyrosine residue; Pi, inorganic phosphate.

It was found that the self-phosphorylation of the protein Wzc attenuates its activity and reduces the level of EPS in *E. coli* [45]. Our results indicate that phosphorylation of Wzc in *G. diazotrophicus* strain Pal5 results in the inhibition of EPS biosynthesis. Here, we present evidence for a novel intercellular signalling system that controls the production of EPS by acting at the protein modification level in *G. diazotrophicus* Pal5, namely tyrosine phosphorylation. Our evidence indicates that the extracellular domain of the Wzc component is a receptor that directly recognizes EPS and thereby regulates kinase activity by the periplasmatic component Wzc. The EPS, as a short-range signalling molecule for cells, acts in the developing biofilm that triggers a positive feedback loop that stimulates EPS production (Figure 7).

The results suggest that in the *G. diazotrophicus* strain Pal5, EPS reacts with the extracellular membrane component of the Wzc receptor to regulate its kinase activity on a first glycosyltransferase. In the MGD mutant, the Wzc is inactivated by self-phosphorylation due to the privation of EPS (Figure 4 and Figure 5). Therefore, the presence of EPS inhibits self-phosphorylation and promotes the phosphorylation of a glycosyltransferase instead, thus stimulating EPS production [46]. In gumD glycosyltransferase, any possible polar effect would only affect the nearby genes (gumM, gumC, gumH, aceG and gumB) that are all predicted to be involved in the same process of EPS production, in which gumD catalyses the first step [16].

Because Wzc may be related to the production of exopolysaccharides in bacteria, we propose that this autoregulation that occurs in *G. diazotrophicus* may be a hallmark evolutionary feature in proteobacteria. The findings of this study, which are based on the presence of an EPS detection process to control EPS production, suggests that under exponential growth conditions, when the population is dominated by individual cells, EPS production by a small number of individual chains may be too scarce to allow EPS to reach sufficiently high levels to stimulate its own production through the kinase BY detection system [23]. Similar results were observed by Schwechheimer and colleagues [47], who demonstrated that purified EPS could stimulate its own production, therefore corroborating our study. The phosphorylation of Wzc suppresses EPS synthesis, whereas EPS-stimulated the phosphorylation of glycosyltransferase (gumD) mediated by Wzc promoting EPS production.

Regarding the relative expression of *wzc* in *G. diazotrophicus* strain Pal5 and in MGD and MWzc grown in different carbon sources, a one-off response to Pal5 was observed. However, there was an increase in the expression of the *wzc* gene when the Pal5 strain was grown in the presence of mannitol and was partially restored in the presence of exogenous EPS. These data suggest that the gene expression pattern can be correlated with the different carbon sources that are present in the growth medium. These data corroborate those found by Meneses et al. [16], who observed very similar exopolysaccharide production when the wild type and mutant of strain Pal5 were grown in different carbon sources. This fact indicates that the higher the presence of exopolysaccharides in the cell, the more the expression levels of *wzc* (GDI2549) in *G. diazotrophicus* strain Pal5. Relative expression was observed when the MGD mutant grew in the presence of glucose, fructose, sucrose, and mannitol, pointing to possible autophosphorylation activity. The wzc gene expression profile results show that EPS does not regulate the expression of the wzc gene. However, EPS directs the BY kinase activity since, in the absence of EPS, the Wzc protein auto-phosphorylates, while in the presence of EPS, it phosphorylates the first glycosyltransferase in the system.

We experimentally demonstrated that the Wzc is rendered inoperative by self-phosphorylation and that exopolymers from *G. diazotrophicus* inhibit self-phosphorylation, thus allowing the kinase to act on other target proteins in the EPS biosynthesis pathway, such as glycosyltransferases. Therefore, EPS is commonly found in bacterial biofilms and is a regulating polymer that promotes its own synthesis. Hence, in addition to having a structural function within bacterial physiology, it would be interesting to investigate whether the EPS produced by the *G. diazotrophicus* Pal5 strain also has other functions in plant signalling.

## 5. Conclusions

*G. diazotrophicus* exopolysaccharide plays a critical role in several important biological processes, including adherence, interactions between bacteria and host plant cells, and resistance to host immunity. Here, we demonstrate that the self-regulation properties of Wzc from *G. diazotrophicus* Pal5 underscore the BY kinases-like proteins that are potentially involved in the polymerisation complex, determining the role of EPS in modulating self-phosphorylation in the biosynthesis of exopolysaccharides on the surface of *G. diazotrophicus* Pal5 cells. Wzc-mediated self-regulation may be a widespread feature of the exopolysaccharide production control in this endophytic diazotrophic bacterium, which is commonly associated with sugarcane plants.

## Figures and Tables

**Table 1:** Bacterial strains and primers used in the work.

Strains and Primers	Characteristics or Sequences	Reference
Pal5	Wild-type, Amp^S^, Km^S^, Tc^S^, EPS^+^	[1]
MGD	gumD::Tn5, Km^R^, gumD^−^, EPS^−^	[16]
MWzc	wzc::Tn5, Km^R^, wzc^−^, EPS^−^	This study
ΔW_Pal5	His6-tagged Wzc recombinant protein of Pal5	This study
ΔW_MGD	His6-tagged Wzc recombinant protein of MGD	This study
ΔW_MWzc	His6-tagged Wzc recombinant protein of MWzc	This study
wzc-sense wzc-antisense	5′-GACCTGGCCAATATGTTCGT-3′ 5′-ATCAGCAGCTTCTTGCGATT-3′	This study
ExpWzcN1-sense ExpWzcN1-antisense	5′-GACCTGGCCAAAATATGTTCGT-3′ 5′-ATCAGCAGCTATATGTTCGT-3′^−^	This study
M13GW-sense M13GW-antisense	5′-GTAAAACGACGGCCAG-3′ 5′-AGGAAACAGCTATGAC-3′	This study
attB1	5′-GGGGACAAGTTTGTACAAAAAAGCAGGCT-3′	Invitrogen
attB2	5′-GGGGACCACTTTGTACAAGAAAGCTGGGT-3′	
23S-sense 23S-antisense	5′AAAGCCGGATCAATCCGTTA3′ 5′AAGCCGTAGTCGATGGAAAC3′	[20]
RTwzc-sense RTwzc-antisense	5′GGGGAAATCGAACAGTTGCG3′ 5′CGGGCGCGCGGTCCGC3′	This study

*gumD* encodes a glycosyltransferase I, *wzc* encodes a tyrosine kinase, Amp (ampicillin), Km (kanamycin), Tc (tetracycline), ^S^ Sensitive, ^R^ resistant, + presence and – absence.

## Data Availability

Not applicable.
